# Effect of Complexation with Arabinogalactan on Pharmacokinetics of “Guest” Drugs in Rats: For Example, Warfarin

**DOI:** 10.1155/2013/156381

**Published:** 2013-12-26

**Authors:** Mikhail V. Khvostov, Alexander A. Chernonosov, Tatjana G. Tolstikova, Marat F. Kasakin, Olga S. Fedorova, Alexander V. Dushkin

**Affiliations:** ^1^N. N. Vorozhtsov Novosibirsk Institute of Organic Chemistry, Siberian Branch of the Russian Academy of Sciences, Academition Lavrentiev Avenue 9, Novosibirsk 630090, Russia; ^2^Institute of Chemical Biology and Fundamental Medicine, Siberian Branch of the Russian Academy of Sciences, Academition Lavrentjev Avenue 8, Novosibirsk 630090, Russia; ^3^Institute of Solid State Chemistry and Mechanochemistry, Siberian Branch of the Russian Academy of Sciences, Kutateladze Street 18, Novosibirsk 630128, Russia

## Abstract

A pharmacokinetic study of the warfarin (WF) : arabinogalactan (AG) complex with the 1 : 10 mass ratio after its intragastric introduction to Wistar rats at a dose of 5 mg/kg (WF dose in the complex was 0.5 mg/kg) once a day for three days was conducted. It was found that *C*_max_, *T*_1/2_, and AUC of WF in the complex form were lower than after the introduction of blank WF at the same dose, but its elimination (Cl, MRT) was much faster. Significant accumulation (*C*_min_) and an abrupt increase in plasma concentration after the third introduction were observed for blank WF, whereas the complex showed a much more moderate increase in concentration at this point. However, despite obvious differences in pharmacokinetic parameters, the efficacies of both agents were virtually identical; the complex differed from blank WF by only 15%. This value is rather insignificant and does not impair its anticoagulant activity. Thus, we can conclude that introduction of the WF : AG complex is safe in terms of reduction of the bleeding risk and accumulation.

## 1. Introduction

The problems of drug bioavailability and safety are highly urgent in modern medicine and pharmacology [1, 2]. They could be solved by creating novel drugs, but it is a very costly way in terms of money and time. The route of a drug to the pharmaceutical market could be delayed by a decade and unfortunately it is not secured against clash with undesired side effects. Thereby, it is much easier to deal with the known drugs that have already been proven to be effective and for which the major side effects are known. It is possible to create novel drug forms aimed at improving bioavailability, decreasing toxicity, and meanwhile preserving efficacy for this type of drugs. Sometimes the initial drug in these forms can reveal a previously unknown effect or enhance the low-grade pharmacological effect [3]. In the modern pharmaceutical industry, there are a lot of ways to do so. One of them employs various synthetic or plant compounds that can form the “host-guest-” type compounds, where a drug molecule acts as a “guest” [2, 4]. We propose to use plant polysaccharide, arabinogalactan, which is found in woody tissue of *Larix sibirica* and *Larix gmelinii* at high amounts, as a “host” molecule in the present study [5]. It has previously been demonstrated that this compound can form complexes with various drugs, resulting in improved water solubility of the initial drug, preservation of the required pharmacological activity, and often the reduction of effective dose [3, 6]. One of these successful examples is water-soluble AG complex with warfarin, which is widely used as an indirect oral anticoagulant prescribed for a long time to patients with different cardiovascular diseases and after complicated surgeries [7, 8]. The new complex was found to be highly effective; however, its anticoagulant activity was not as sharp as that of warfarin [9]. These data suggested that the complex is safer as compared to blank warfarin, since smoother blood dilution can reduce the risk of bleeding, which is one of the major side effects of warfarin [7, 8, 10, 11]. In order to clarify this hypothesis, we conducted a pharmacokinetic study of the WF : AG complex intragastrically introduced to rats for several days.

## 2. Materials and Methods

### 2.1. Chemicals

WF (A2250) was purchased from Sigma-Aldrich. AG was obtained at the Laboratory of Wood Chemistry (Professor V. A. Babkin), A. E. Favorsky Institute of Chemistry, SB RAS. The WF : AG complex at a weight ratio of 1 : 10 was synthesized using the mechanochemical method at the Institute of Solid State Chemistry and Mechanochemistry, SB RAS [12].

### 2.2. Animals

The study involved female Wistar rats (190–210 g). The animals were obtained at the laboratory of experimental animals of the Institute of Cytology and Genetics (SB RAS, Novosibirsk). All manipulations were performed in full accordance with the rules and principles of humane animal treatment.

The animals (6 per group) had stayed hungry for 12 h before the dosing. All compounds were dissolved in distilled water. Compounds were administered at the same time intragastrically once a day during 3 days at a dose of 0.5 mg/kg for WF and 5 mg/kg for the complex (WF dose 0.5 mg/kg). Tween 80 was used additionally to dissolve WF. Blood was collected daily from tail vessels into the tubes with 3.8% sodium citrate (9 : 1) 1, 4, 6, 12, and 24 h after WF or WF : AG administration.

In order to determine the prothrombin time (PT) 24 hours after the last introduction, blood was drawn from the neck vessels (sodium citrate was used as an anticoagulant, with the blood/citrate ratio being 9 : 1). The drawn blood was centrifuged at 3000 rpm for 15 min. Blood plasma was subsequently collected. PT was studied via spectrophotometric methods, using the standard set for the PT definition (Tekhnologia-standard, Russia) on a clot 1A coagulometer (Hospitex Diagnostics S. A.).

### 2.3. Preparation of Standard Stock Solutions

The standard stock solutions of racemic warfarin (1 mg/mL) and internal standard (29.2 mg/mL) of chlorowarfarin (I.S.) were prepared by dissolving the respective compounds within 50% acetonitrile in water. A series of calibration solutions of racemic warfarin were prepared by adding the appropriate volumes of the standard stock solution to drug-free rat plasma. Additionally, three levels of quality control (QC) samples (3, 7, and 18 mg/mL of racemic warfarin) were prepared. The plasma standards and QC samples were aliquoted, stored, and treated the same way as the rat plasma samples. The calibration plasma solutions contained 0.2, 0.5, 1, 2, 4, 6, 8, 10, 15, and 20 *μ*g/mL of warfarin and 2.9 *μ*g/mL of I.S. The calibration curves were constructed via linear regression analysis of peak-area ratios of the drug to the internal standard versus the respective concentration of the calibration plasma solutions. Three replicate injections of standards were performed at each concentration. The linear assay range was 0.2–20 *μ*g/mL for warfarin (*r* ^2^ > 0.99). No interference in the blank sample was noted.

### 2.4. Samples Preparation

The current extraction method was based on the “metabolomics method” [13]. Whole blood samples (200 mL) collected 1, 4, 6, 12, 24, 25, 28, 30, 36, 48, 49, 52, 54, 60, 72, 78, and 96 h after the administration of WF or the WF : AG complex were centrifuged for 10 min at 3000 rpm in order to separate the plasma fraction. 10 mL of I.S. was added to each sample at a concentration of 29.2 *μ*g/mL. 285 mL of ice-cold 0.2% formic acid in H_2_O was added to 100 mL of sample plasma in 2 mL tubes. The tubes were vortex mixed for 10 s, and 1.5 mL of acetonitrile was added as an extraction solvent. The solutions were shaken for 30 min at 37°C at 450 cycles per min in the vertical position using an Eppendorf mixer 5432 (Eppendorf, USA). After centrifugation for 5 min at 13000 rpm, 1.5 mL of the upper organic layer was transferred in order to clean the tube and evaporated to dryness in a speed vacuum concentrator. The precipitate was resolved in 200 mL of 0.01% formic acid in acetonitrile : water (1 : 1) solvent and injected for MS/MS analysis.

### 2.5. MS/MS Analysis of Warfarin

Warfarin concentrations in all plasma samples were analyzed via the MS method using the Agilent 6410 QQQ mass spectrometer (Agilent Technologies, USA). Quantitative analysis was performed in the positive-ion detection mode (ESI) and set up in the multiple reaction-monitoring mode (*m*/*z*  307.0 → 160.9 for warfarin and 340.9 → 283.8 for I.S.) with the total running time of 2.5 min for each sample. Nitrogen was used as a desolvation and nebulizer gas (gas flow rate was 3.5 L/min). The source and desolvation temperatures were set at 200 and 350°C, respectively. The capillary voltage was 2.5 kV. The water-acetonitrile mixture (1 : 1) was used as the mobile phase with 1 mL/min flow rate. Each sample was analyzed three times.

### 2.6. Pharmacokinetic Data Analysis

A noncompartmental analysis (bear version 2.5.3 (http://pkpd.kmu.edu.tw/bear/) as a package for R version 2.12.0) was used for calculations with the following setups: multiple-dose study with warfarin at a dose of 1 mg, Tau 24 h, and manual selection of the exact 3 data points. The maximum (*C*_max⁡_) and minimum (*C*_min⁡_) plasma concentrations and time to reach *C*_max⁡_  (*T*_max⁡_) were obtained from the observed time-*C*_*p*_ profile. The linear trapezoidal rule method was used to calculate AUC_0–*t*_ (AUC from time 0 to the last measurable *C*_*p*_). The extrapolated AUC (from time of the last measurable *C*_*p*_ to infinity) will be estimated from the last measurable *C*_*p*_ divided by *λ*_z_. AUC_0−inf⁡_ (AUC from time 0 to infinity) is equal to AUC_0−*t*_ plus the extrapolated AUC (AUC_*t*−inf⁡_). The other calculated parameters include the average plasma concentration (*C*_av_), clearance (Cl/F), mean residence time (MRT), apparent elimination half-time (*T*_1/2_), apparent volume of distribution (*V*_*d*_/F), area under the concentration times time versus time curve (AUC), and terminal elimination rate constant (*λ*_z_). All results were expressed as the mean ± SD.

## 3. Results and Discussion

The complex formation was confirmed by differential scanning calorimetry and increased aqueous solubility of WF [9].

**Figure 1:**
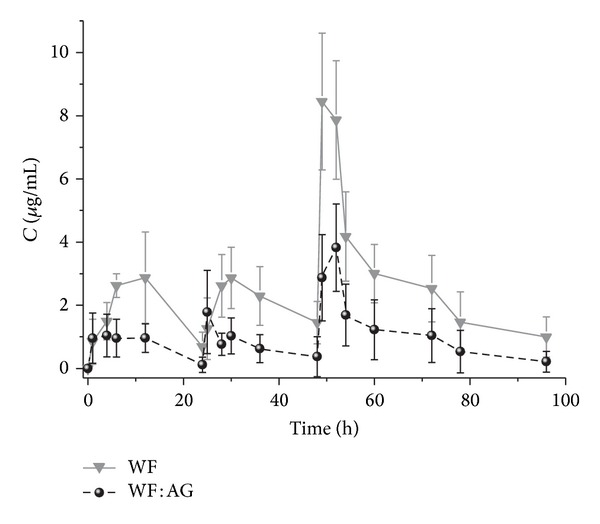
The mean plasma concentration-time profile of WF : AG (1 : 10 ratio) and blank WF after repeated oral administration at a dose of 5 mg/kg (WF dose is equal to 0.5 mg/kg) and 0.5 mg/kg, respectively.

Rats are capable of bearing high single oral doses of WF; however, repeated introduction at doses over 1 mg/kg/day become lethal [14]. Hence, WF dose of 0.5 mg/kg/day was selected in this study. The data obtained is shown in Figure 1 and Table 1.

It is clear from the data that the pharmacokinetic curves for both compounds are analogous; the concentration-time profile for WF during the first 24 hours is comparable to the data available in the literature [15]. However, plasma concentration (*C*_max⁡_) of WF after its intragastric introduction in the form of the complex with AG was 60% lower as compared to the introduction of blank WF. Its elimination occurred much faster and leads to 12 hours lower *T*_1/2_ and 70% higher Cl values. The most significant differences can be observed after the third introduction (48 h after the first introduction) of agents. Clear accumulation (*C*_min⁡_1.31 ± 0.13 *μ*g/mL versus 0.17 ± 0.30 for the complex) and abrupt increase in plasma concentration of blank WF were observed 1 h after the introduction (point at 49 h). No such significant concentration changes were observed for the complex. Moreover, the *T*_max⁡_ for both agents was equal, attesting to the fact that there was no delay in absorption of WF in the complex form. Thus, a conclusion can be drawn that the water-soluble form of WF (complex) enters the blood stream and remains more hydrophilic over blank WF, thus favoring its faster elimination.

**Figure 2:**
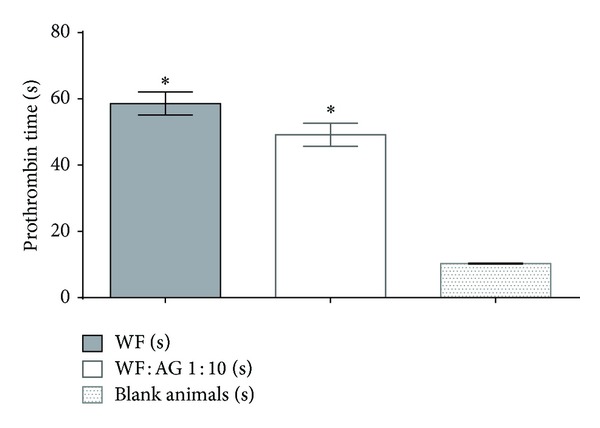
Prothrombin time duration 24 hours after the last oral introduction of WF : AG 1 : 10 at dose 5 mg/kg and WF at dose 0.5 mg/kg.

The primary pharmacological effect of WF is its anticoagulant activity, which is evaluated by prothrombin time duration, the common test for such compounds [16]. We have evaluated it in the present study in order to compare the efficacy of the aforedescribed agents. The anticoagulant activity of the WF : AG complex was found to be only 15% lower than that of WF; meanwhile, the complex retains its efficacy after blood dilution (Figure 2).

## 4. Conclusions

A pharmacokinetic study of the WF : AG complex with the 1 : 10 ratio after its intragastric introduction to Wistar rats at a dose 5 mg/kg (WF dose in the complex was 0.5 mg/kg) once a day for three days was conducted. It was found that *C*_max⁡_, *T*_1/2_ and AUC of WF in the complex form were lower as compared to these values after the introduction of blank WF at the same dose, but its elimination (Cl, MRT) was found to be much faster. In the case of blank WF, significant accumulation (*C*_min⁡_) and an abrupt increase in plasma concentration after the third introduction was observed, whereas the complex exhibited a significantly more moderate increase in concentration at this point. However, despite the obvious differences in pharmacokinetic parameters, the efficacies of both agents were virtually identical, and the complex differed from blank WF only by 15%, which is rather insignificant and does not impair its anticoagulant activity. Thus, it can be concluded that the introduction of WF in its complex with AG is safer in terms of reduction of bleeding risk and accumulation.

## Figures and Tables

**Table 1:** Pharmacokinetic parameters of WF and 1 : 10 WF : AG.

	1 : 10 WF : AG	Warfarin
*λ*_z_, 10^−2^ h^−1^	6.70 ± 2.30*	3.00 ± 0.20
*C*_max⁡__ss, *μ*g/mL	3.85 ± 1.40*	9.12 ± 1.40
*C*_min⁡__ss, *μ*g/mL	0.17 ± 0.30*	1.31 ± 0.13
*T*_max⁡__ss, h	51.40 ± 1.30	50.50 ± 1.70
*C*_av_, *μ*g/mL	1.29 ± 0.38*	5.42 ± 1.89
Cl/F, mL/h	2.98 ± 1.25*	0.84 ± 0.10
*V*_*d*_/F, mL/kg	36.93 ± 3.70*	26.23 ± 5.67
*T*_1/2_(z), h	11.64 ± 4.85*	23.41 ± 1.40
AUC(tau)ss, *μ*g h/mL	30.95 ± 9.19*	130.25 ± 45.25
AUMC(tau)ss, *μ*g h^2^/mL	310.25 ± 149.79*	2501.23 ± 313.23
MRT, h	12.45 ± 3.08*	16.55 ± 0.58

*p < 0.05 from warfarin.
